# Retraction Note: Investigating the Heteronjunction between ZnO/Fe_2_O_3_ and g-C_3_N_4_ for an Enhanced Photocatalytic H_2_ production under visible-light irradiation

**DOI:** 10.1038/s41598-024-58322-1

**Published:** 2024-04-02

**Authors:** Na Mao

**Affiliations:** 1 Shaanxi Key Laboratory for Advanced Energy Devices, Key Laboratory for Macromolecular Science of Shaanxi Province, School of Materials Science and Engineering, Shaanxi Normal University, Xi’an, 710062 Shaanxi People’s Republic of China; 2 College of Chemistry and Materials, Weinan Normal University, Weinan, 714099 Shaanxi People’s Republic of China

Retraction of: *Scientific Reports* 10.1038/s41598-019-48730-z, published online 27 August 2019

The Editors have retracted this Article.

After publication the Editors were made aware of concerns about the data presented. Specifically:

- There are four similar XRD patterns in Figure 2a (7-ZnO/Fe ~ 2 ~ O ~ 3 ~ /g-C ~ 3 ~ N ~ 4 ~ , 5-ZnO/Fe ~ 2 ~ O ~ 3 ~ /g-C ~ 3 ~ N ~ 4 ~ , 3-ZnO/Fe ~ 2 ~ O ~ 3 ~ /g-C ~ 3 ~ N ~ 4 ~ and 1-ZnO/Fe ~ 2 ~ O ~ 3 ~ /g-C ~ 3 ~ N ~ 4 ~);
- The XRD patterns appear to be similar between Figure 2a and Supplementary Figure S8a.

In addition, there are similarities between figures in this Article and in another publication^1^ by the same author group. Specifically:

- Figure 2b appears to be similar to Figure 1b in^1^ where different compounds are used;
- Figure 6c appears to be similar to Figure 5c in^1^ where different compounds are used.

The Editors therefore no longer have confidence in the reliability of the data presented.

Na Mao agrees with this retraction.
